# Case Report: Next-Generation Sequencing in Diagnosis of Pneumonia Due to *Pneumocystis jirovecii* and Cytomegalovirus in a Patient With HIV Infection

**DOI:** 10.3389/fmed.2021.653294

**Published:** 2021-03-29

**Authors:** Yirui Xie, Bing Ruan, Lingxiao Jin, Biao Zhu

**Affiliations:** ^1^State Key Laboratory for Diagnosis and Treatment of Infectious Diseases, The Department of Infectious Diseases, National Clinical Research Center for Infectious Diseases, Collaborative Innovation Center for Diagnosis and Treatment of Infectious Diseases, School of Medicine, The First Affiliated Hospital, Zhejiang University, Hangzhou, China; ^2^Department of Infectious Diseases, People's Hospital of Pujiang County, Jinhua, China; ^3^School of Medicine, Pujiang Branch of the First Affiliated Hospital, Zhejiang University, Hangzhou, China

**Keywords:** next-generation sequencing, *Pneumocystis jirovecii*, cytomegalovirus, bronchoalveolar lavage fluid, pneumonitis, HIV

## Abstract

**Background:** Pulmonary infections remain a significant cause of morbidity and mortality in immunocompromised patients. The pathogens spectrum of pulmonary infection that can affect patients with human immunodeficiency virus (HIV) is wide such as bacterial, fungal, viral, parasitic organisms, and so on. The risk of multi-pathogenic pneumonia is higher in HIV-infected patients. However, the fast and accurate diagnosis of multi-pathogenic pneumonia is challenging because of the limitations of current conventional tests.

**Case Presentation:** Here, we report a case of pneumonia due to *Pneumocystis jirovecii* and cytomegalovirus (CMV) in a 22-year-old male with newly diagnosed HIV infection. Blood tests revealed a low CD4 count, a chest computed tomography (CT) scan showed extensive ground-glass opacities in the bilateral lung with multiple cavity lesions in the left upper lung. Microscopic examination of stained sputum and bronchoalveolar lavage fluid (BALF) smear specimens did not find any pathogens. There was also no evidence of pathogens known to cause pneumonia in bacteria and fungi culture tests and virus antibodies such as EBV, CMV, and COVID-19. The nucleic acid of CMV in blood was reported by quantitative PCR. Next-generation sequencing (NGS) analysis of BALF specimens identified a large number of *P. jirovecii* and CMV reads, and confirmed the diagnosis of pneumonia due to *P. jirovecii* and CMV. Following the patient's treatment with anti-PCP and anti-CMV, the patient was cured and discharged.

**Conclusions:** This case highlights the combined application of NGS in the clinical diagnosis of multi-pathogenic pneumonia in an HIV-infected patient. NGS is proposed as an important adjunctive diagnostic approach for identifying pathogens of multi-pathogenic pneumonia in HIV-infected patients.

## Introduction

Pulmonary infections can be caused by a variety of microbes including bacteria, fungi, and viruses, which can be severe and fatal particularly among immunocompromised individuals (1–3). The risk of mixed pulmonary infection is high in immunocompromised patients such as patients with HIV infection (4, 5). Compared to patients with monomicrobial pulmonary infection, patients with mixed pulmonary infection may have a more severe clinical manifestation and are difficult to diagnose. The diagnosis of multi-pathogenic pneumonia must be as fast and accurate as possible, because severe clinical manifestation and combined treatment have many potential side effects. However, fast and accurate diagnosis of multi-pathogenic pneumonia is challenging because of the limitations of current conventional tests (6). Occasionally, we still cannot draw a correct conclusion according to these tests. Next-generation sequencing (NGS), a highly sensitive method for analyzing the microbiome, could provide additional valuable information for the detection of pathogens. Here, we report a patient with newly diagnosed HIV infection who developed multi-pathogenic pneumonia revealed by NGS of bronchoalveolar lavage fluid (BALF).

## Case Report

A 22-year-old Chinese male form Zhejiang province initially presented to the local community hospital. He complained of 10 days of intermittent fever with a temperature of up to 38.7°C and 3 days of progressive dyspnea and dry cough. He was given moxifloxacin at the local community hospital. However, he contracted chest thrush after using moxifloxacin, so this treatment was stopped and he was given ambroxol hydrochloride. Due to a history of male sexual partners and weight loss, an HIV test was obtained which returned positive. Then he came to our institution, the infectious disease department of the First Affiliated Hospital, School of Medicine, Zhejiang University, and hospitalized.

**Figure 1:**
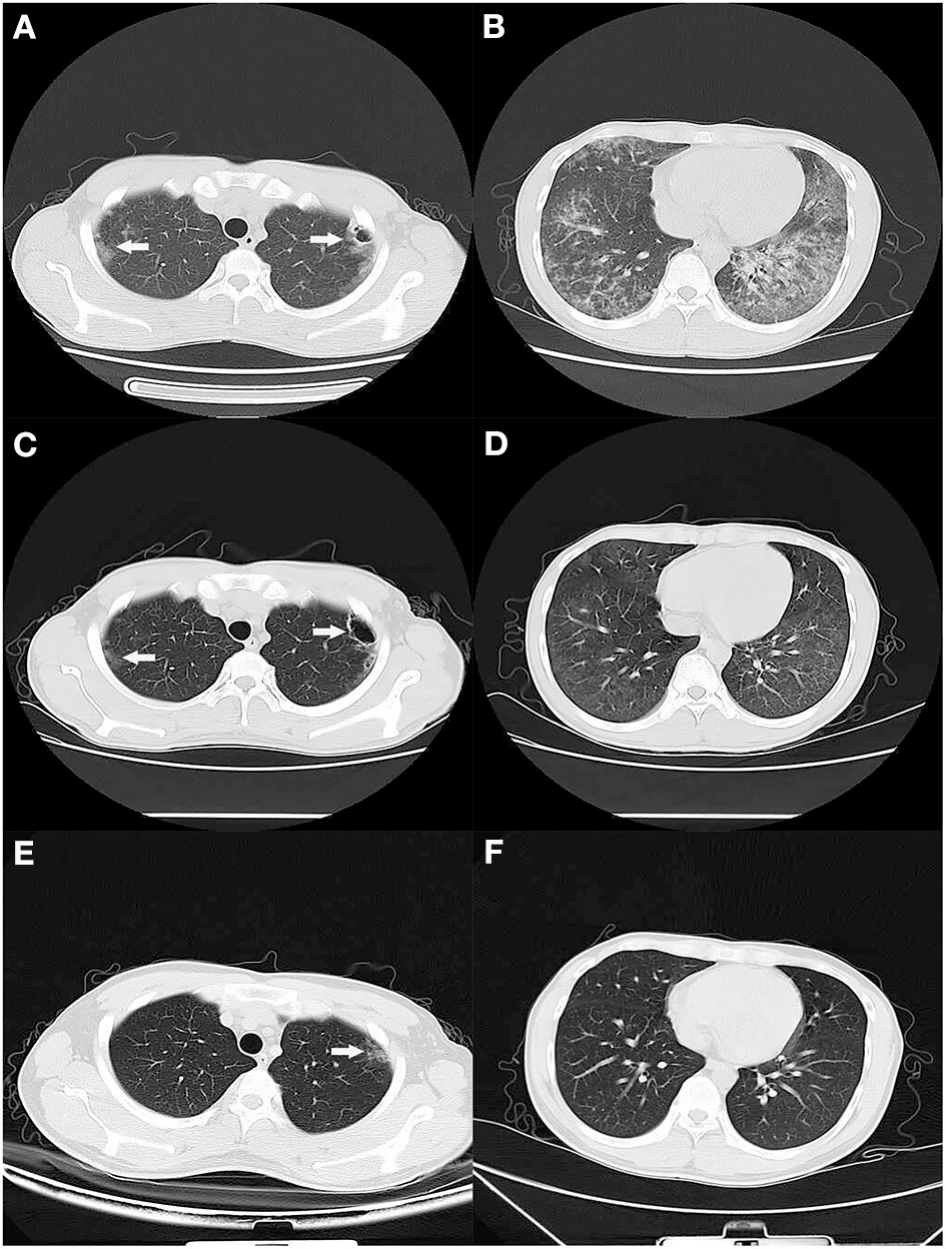
CT scan of the lung. **(A,B)** On the 1st day of admission, a CT scan showed diffuse bilateral ground-glass opacities with thin-walled cavity lesions (white arrow) in the left upper lung. **(C,D)** After 2 weeks of treatment, a CT scan showed that lesions were significantly absorbed except for a faint patchy opacity in the lateral segment and thin-walled cavity lesions (white arrow) in the left upper lung. **(E,F)** Two months later, a CT scan exhibited no abnormal finding except for a faint patchy opacity (white arrow) in the left upper lateral segment.

Upon presentation at our institution, he still had fever, dyspnea, fatigue, cough with a small amount of pale sputum, and occasionally pectoralgia and dyspnea during activity. Physical examination found a body temperature of 37.4°C, blood pressure of 113/50 mmHg, a pulse of 115 beats per minute, a respiration rate of 23 breaths per minute, and SpO_2_ 93% using fingertip pulse oximetry. The bilateral lung presented moist rales. The patient did not have a skin rash. The laboratory results were as follows: white blood cell count 7.7 × 10^9^/L, with a neutrophil ratio of 65.7%, hemoglobin 133 g/L, and platelet count 365 × 10^9^/L; C-reactive protein (CRP) 8.5 mg/L; erythrocyte sedimentation rate (ESR) 5 mm/h; and procalcitonin 0.08 ng/mL. Blood biochemical index results showed that lactate dehydrogenase content increased to 401 U/L (reference range 150–250 U/L), and -hydroxybutyrate dehydrogenase content increased to 326 U/L (reference range 72–182 U/L). The 1,3-beta-D-glucan test (G test) result (251.64 pg/ml) was positive, while galactomannan test (GM test) (0.1 μg/L) and cryptococcus capsular antigen test results were negative. His CD4 count was 16 cells/μL. Arterial blood gas analysis (with O_2_ 3 L/min *via* nasal catheter) showed pH 7.51; pCO_2_ 27 mmHg; pO_2_ 98 mmHg; HCO_3_ −21.9 mmol/L; SBE −0.8 mmol/L; lactate 0.8 mmol/L; and O_2_ saturation 99%. A chest computed tomography (CT) scan showed extensive ground-glass opacities in the bilateral lung with multiple thin-walled cavity lesions in the left upper lung (Figures 1A,B). The IgG and IgM antibodies of coronavirus disease 2019 (COVID-19) were negative. The nucleic acid detection of COVID-19 was negative. Nucleic acid detection of common respiratory pathogens (including Mycoplasma pneumoniae, Chlamydia pneumoniae, adenovirus, respiratory syncytial virus, parainfluenza virus, and influenza A and B) was negative. The IgG antibodies of Epstein-Barr virus (EBV) and human *cytomegalovirus* (CMV) were positive, while the IgM antibodies of EBV and CMV were negative. The nucleic acid of CMV in the blood was reported at 1.86 × 10^4^ copies/mL by quantitative PCR. Mycobacterium tuberculosis (MTB) was negative based on blood test with T-SPOT.TB as well as acid-fast staining of sputum smear and GeneXpert. The blood, sputum, and urine smear and culture of bacteria and fungi were negative. We also sent the sputum sample to perform Gomori-Grocott methenamine silver nitrate staining (GMS) for *Pneumocystis jirovecii* detection and obtained a negative result. Given the patient's hypoxia and demonstration of diffuse ground-glass opacities on CT of the chest, a clinical diagnosis of *P. jirovecii* pneumonia (PCP) was made. Oral trimethoprim-sulfamethoxazole (TMP-SMX, TMP 0.24 g /SMX 1.2 g, q8h), intravenous clindamycin (0.6 g, q12h), and methylprednisolone (40 mg daily) were administered, which resulted in the improvement of general conditions and abatement of fever and dyspnea in 3 days.

To verify pathogen-induced pneumonia, the patient underwent a bronchoscopy examination with a collection of BALF specimens on day 5 of admission. The BALF was sent to the laboratory for conventional tests and an NGS test [using the MGISeq 2000 platform (MGI Tec Co. Ltd, Shenzhen, China)]. The BALF Gram stain and acid-fast stain were negative. The G test and GM test of BALF were negative. In addition, a BALF culture of fungal and bacteria showed no organisms. Microbiological tests of BALF were negative for MTB by the smear and GeneXpert MTB/RIF assay. Two days later, the NGS results showed that *P. jirovecii* (1,000,078 reads) and CMV (4,192 reads) were found in the BALF. The other microorganisms detected by NGS were bacteria and are listed in Table 1, but they were not regarded as responsible for the invasive pulmonary infection. Based on the report, the diagnosis of multi-pathogenic pneumonia due to PCP and CMV was made in this patient, and intravenous ganciclovir (0.25, Q12H) was started.

**Table 1:** The other microorganisms detected by next-generation sequencing (NGS) in the BALF of the case.

**Genus**		**Species**	
**Name**	**Sequence** ** number^#^**	**Name**	**Sequence** ** number^#^**
*Staphylococcus*	172	*S. epidermidis*	130
*Prevotella*	105	*Prevotella melaninogenica*	65
*Streptococcus*	44	*S. sanguis*	15
		*S. mitis*	3
*Cutiibacterium*	13	*C. acnes*	7
*Rothia*	10	*R. mucilaginosa*	10
*Veillonella*	7	*V. atypical*	4
*Moraxella*	5	*M. osloensis*	5

# *The sequence number of the strict comparison of the microorganism detected at the level of genus/species*.

After 2 weeks of hospitalization, his symptoms disappeared, general conditions returned to normal, and laboratory findings improved. The chest CT scan showed that lesions had been significantly absorbed except for a faint patchy opacity in the lateral segment and thin-walled cavity lesions in the left upper lung (Figures 1C,D). So, the patient began antiretroviral therapy with Truvada (emtricitabine and tenofovir disoproxil fumarate tablets, 500 mg QD) and dolutegravir (50 mg QD). He was discharged to complete 1 week of TMP-SMX and 2 weeks of oral ganciclovir (500 mg, three times daily) at home, and then switch to prophylactic doses of TMP-SMX until CD4 count recovery. At his 2-week follow-up visit, he had no symptoms. At his 2-month follow up, he appeared healthy with no symptoms and a chest CT scan exhibited no abnormal findings except for a faint patchy opacity in the left lateral segment (Figures 1E,F).

## Discussion

With the widespread use of highly active anti-retroviral therapy (HAART), the incidence of AIDS-related opportunity infections has declined dramatically (7–9). However, a rapid growth of HIV-infected cases, poor adherence to medications, and the emergence of virological and immunological failure have caused infections to thrive (10, 11). The risk of multi-pathogenic pneumonia is higher in HIV-infected patients and the spectrum of pathogens is broad including bacterial, fungal, viral, and parasitic organisms (7, 12). So, consideration of the possibility of a multi-pathogenic pneumonia is essential in HIV-infected patients and early bronchoscopy is recommended. Conventional tests including microscopy, specific antigen or antibody detection, PCR amplification, and culture have been utilized in clinical work. However, these conventional tests have limitations in terms of sensitivity, speed, spectrum for pathogen detection, and simultaneous multi-pathogen detection (6, 12). So, the prompt appropriate diagnosis of multi-pathogenic pneumonia in HIV-infected patients is still challenging.

Next-generation sequencing (NGS) is a highly sensitive, culture-independent, and unbiased method that can identify all potential known and new or unexpected pathogens (13, 14). NGS has been successfully used as a diagnostic tool for various infectious diseases in immunocompromised hosts (5, 15). Its application to diagnose multi-pathogenic pneumonia due to PCP and CMV in HIV-infected patients has never been reported until now. Here we report a case of PCP and CMV multi-pathogenic pneumonia diagnosed by NGS of BALF following negative results in multiple conventional diagnostic tests.

PCP is serious and sometimes critical infections caused by *P. jirovecii* occur in immune-suppressed patients (16, 17). Up until now, the definitive diagnosis of PCP is confirmed upon finding *P. jirovecii* in respiratory secretion, tissue, or a BALF sample. A Wright-Giemsa stained smear was the most common conventional diagnostic method in the past few decades and other methods such as methenamine silver and cresyl echt violet fluorescence staining and nucleic acid amplification testing may further increase the detection sensitivity. Despite high incidence of PCP in HIV-infected patients, the diagnosis of PCP remains challenging due to its non-specific signs and symptoms and inadequate performance of conventional diagnostic methods (18). CMV pneumonitis is uncommon, and its diagnosis is challenging as the clinical manifestations and radiological features of CMV pneumonia are also non-specific (7, 10, 18, 19). The challenges of prompt appropriate diagnosis of PCP and CMV-mixed pulmonary infection are well-reflected in the present case report. Consistent with our patient, HIV-infected patients with a CD4 count <100 cells are at higher risk for CMV infection and/or reactivation. As a result of viral shedding, it is common for CMV to present in the BALF of HIV-infected patients; thus, its presence is insufficient for the diagnosis of invasive CMV pneumonitis. The definitive diagnosis of CMV pneumonitis relies on BALF cytology or a lung biopsy demonstrating cells with inclusion bodies, after excluding other etiologies (7, 20). So, CMV pneumonitis was reported in only 5–8% of HIV patients undergoing BALF testing (19). Quantitative polymerase chain reaction (PCR) of CMV viral load has been investigated as a marker of invasive CMV disease, with a sensitivity of 86% and specificity of 87% when the cut-off is applied at >5,000 copies/ml, but mainly in transplant recipients (21–23).

CMV and PCP coinfection has been previously reported in the literature (10). However, there have only been two study reports of 12 cases on the successful use of NGS to diagnose pulmonary infections involving PCP and CMV in BALF or blood or lung biopsy (Table 2) until now (5, 15). The study reported that NGS is more sensitive than conventional methods (non-PCR based). However, the numbers of sequence reads for *P. jirovecii* (5–303,572) and CMV (4–23,282) are highly variable among cases (5, 15). So, the cut-off reads of NGS to be used as a marker of invasive PCP and CMV disease is uncertain. A study suggested that specific reads of *P. jirovecii* ranking among the top 15 or its relative reads proportion in fungi higher than 85% might be satisfactory cut-off values for clinical diagnosis of PCP (18). In patients with PCP, the co-presence of CMV in BALF has traditionally been presumed to be non-invasive and there were no difference in outcome between anti-CMV-treated and CMV-untreated groups (24). However, several reports have highlighted a poor outcome among HIV patients who were co-infected with PCP and CMV (25, 26). A study reported a 2-fold higher mortality at 3 months in patients with PCP who also had CMV cultured from BALF with corticosteroids treatment (27). In our case, the *P. jirovecii* and CMV reads were 1,000,078 and 4,192, respectively, and the patient was treated with corticosteroids. So, additional antiviral therapy with ganciclovir was given and the patient was treated successfully according to the diagnosis based on NGS. This case demonstrates that the addition of anti-CMV therapy may benefit such patients in the right clinical scenario. Furthermore, NGS might be beneficial to HIV-infected patients who develop pneumonia and are more likely to be infected by multiple pathogens. Nevertheless, it is necessary to investigate which cut-off reads should be used as a diagnosis marker of invasive PCP and CMV disease. Multi-pathogenic pneumonia in severely immunocompromised patients presents a diagnostic and management dilemma. Consideration of multiple etiologies in immunocompromised patients is necessary, and early bronchoscopy and NGS are recommended. The resulting interpretation of NGS needs caution, along with other laboratory, radiological, and clinical findings. Furthermore, the application of NGS to clinical diagnosis is still challenging due to barriers in cost-effectiveness and the standardization of the entire process from sample collection to result interpretation (28). Clearly, all these issues need investigation in the future.

**Table 2:** Reports of PCP and CMV cases detected by next-generation sequencing (NGS).

**Patient ID**	**Underlying disease**	**Pulmonary disorders**	**Smear results**	**Culture results**	**Pathology results**	**NGS-based diagnosis (number of unique reads)**	**Reference no**.
No. 1	Immunological anemia	Pulmonary infection	Negative	Negative	Alveolar septal fibrous tissue hyperplasia,	*P. jirovecii (28)*	(5)
		Type I respiratory failure			Inflammatory cell infiltration	*H. cytomegalovirus (72)*	
No. 2	Acute myeloid leukemia	Pulmonary infection	Negative	Negative	Fibrous tissue hyperplasia,	*P. jirovecii (50916)*	(5)
					Inflammatory cell infiltration	*H. cytomegalovirus (10)*	
No. 3	Autoimmune hemolytic anemia	Pulmonary infection	Gram-negative bacilli	Negative	Chronic inflammation of the mucosa, a small amount of neutrophil infiltration, partial alveolar septum widening with fibrous tissue hyperplasia	*P. jirovecii (64), H. cytomegalovirus (23282), Rhizopus microspores (6)*	(5)
No. 4	Acute lymphocytic leukemia	Pulmonary infection	Negative	*P. jirovecii*	Lymphocyte, plasma cell, and neutrophil infiltration	*P. jirovecii (1058), H. cytomegalovirus (154)*	(5)
No. 5	Lymphoma	Pulmonary infection	Gram-positive cocci, Gram-negative bacilli	*Staphylococcus epidermidis*	Alveolar fibrous tissue hyperplasia	*P. jirovecii (303572), H. cytomegalovirus (1626)*	(5)
No. 6	None	Pulmonary infection	Gram-positive cocci	*Acinetobacter baumannii, P. jirovecii*	Inflammatory cell infiltration and interstitial fibrous tissue hyperplasia	*P. jirovecii (4522), H. cytomegalovirus (4), Aspergillus fumigatus (6), Acinetobacter baumannii (338)*	(5)
No. 7	None	Pulmonary infection	Negative	*P. jirovecii*	Inflammatory cell infiltration and interstitial fibrous tissue hyperplasia	*P. jirovecii (104054), H. cytomegalovirus (29)*	(5)
No. 8	Lymphoma	Pulmonary infection	Negative	*P. jirovecii*	Mild hyperplasia of fibrous tissue, a little lymphocyte infiltration	*P. jirovecii (86), H. cytomegalovirus (4)*	(5)
No. 9	Lymphoma	Pulmonary infection	Fungi	Candida	–	*P. jirovecii (427), H. cytomegalovirus (652), Candida albicans (2), Staphylococcus aureus (2)*	(15)
No. 10	Lymphoma	Pulmonary infection	Negative	Negative	–	*P. jirovecii (5), H. cytomegalovirus (28)*	(15)
No. 11	Thymoma; pemphigus	Pulmonary infection	Negative	Negative	–	*P. jirovecii (9), H. cytomegalovirus (187), Nocardia cyriacigeorgica (42)*	(15)
No. 12	Breast cancer; liver cirrhosis; diabetes; renal insufficiency; cardiac insufficiency	Pulmonary infection	Negative	Negative	–	*P. jirovecii (5), H. cytomegalovirus (38), Candida tropicalis (3791), Aspergillus fumigatus (3)*	(15)

*P. jirovecii, Pneumocystis jirovecii; H. cytomegalovirus, human cytomegalovirus*.

## Data Availability Statement

The original contributions generated for the study are included in the article/supplementary material, further inquiries can be directed to the corresponding authors.

## Ethics Statement

Written informed consent was obtained from the patient for the publication of this case report.

## Author Contributions

YX collected data and wrote the manuscript. LJ and BZ analyzed and interpreted patient data. BR and BZ reviewed the manuscript. All authors read through and approved the final manuscript.

## Conflict of Interest

The authors declare that the research was conducted in the absence of any commercial or financial relationships that could be construed as a potential conflict of interest.
